# MSA clustering enhances AF-Multimer’s ability to predict conformational landscapes of protein–protein interactions

**DOI:** 10.1093/bioadv/vbae197

**Published:** 2024-12-06

**Authors:** Khondamir R Rustamov, Artyom Y Baev

**Affiliations:** Laboratory of Experimental Biophysics, Center for Advanced Technologies, Tashkent, 100174, Uzbekistan; Laboratory of Experimental Biophysics, Center for Advanced Technologies, Tashkent, 100174, Uzbekistan; Department of Biophysics, National University of Uzbekistan, Tashkent, 100174, Uzbekistan

## Abstract

**Motivation:**

Understanding the conformational landscape of protein–ligand interactions is critical for elucidating the binding mechanisms that govern these interactions. Traditional methods like molecular dynamics (MD) simulations are computationally intensive, leading to a demand for more efficient approaches. This study explores how multiple sequence alignment (MSA) clustering enhance AF-Multimer’s ability to predict conformational landscapes, particularly for proteins with multiple conformational states.

**Results:**

We verified this approach by predicting the conformational landscapes of chemokine receptor 4 (CXCR4) and glucagon receptor (GCGR) in the presence of their agonists and antagonists. In our experiments, AF-Multimer predicted the structures of CXCR4 and GCGR predominantly in active state in the presence of agonists and in inactive state in the presence of antagonists. Moreover, we tested our approach with proteins known to switch between monomeric and dimeric states, such as lymphotactin, SH3, and thermonuclease. AFcluster-Multimer accurately predicted conformational states during oligomerization, which AFcluster with AlphaFold2 alone fails to achieve. In conclusion, MSA clustering enhances AF-Multimer’s ability to predict protein conformational landscapes and mechanistic effects of ligand binding, offering a robust tool for understanding protein–ligand interactions.

**Availability and implementation:**

Code for running AFcluster-Multimer is available at https://github.com/KhondamirRustamov/AF-Multimer-cluster

## 1 Introduction

Understanding the mechanistic effects of protein–protein interactions (PPIs) requires an in-depth knowledge of the conformational substates that metamorphic proteins can adopt (Henzler-Wildman and Kern 2007). Traditionally, molecular dynamics (MD) simulations have been the most widely used computational method to elucidate the mechanisms of protein actions. MD simulations capture the changes in the conformational space of receptor proteins, which in turn explain the action mechanisms of studied compounds (Latorraca *et al.* 2017). However, MD simulations are computationally intensive and time-consuming, rendering them inefficient for large-scale studies involving diverse protein sets.

Recent advancements in machine learning algorithms have revolutionized computational biology, particularly with the advent of AlphaFold2 (AF2) (Jumper *et al.* 2021), which significantly improved the prediction of single protein structures (Pereira *et al.* 2021). Its variant, AF-Multimer (Evans *et al.* 2022), has shown high accuracy in predicting homo- and hetero-oligomeric protein structures. Despite these advancements, AF2’s initial limitation was its inability to predict different substates of metamorphic proteins (Chakravarty and Porter 2022, Saldaño *et al.* 2022), a challenge that has been recently addressed by various multiple sequence alignment (MSA) clustering approaches (Monteiro da Silva *et al.* 2024, Wayment-Steele *et al.* 2024). Nonetheless, AF2 still struggles to predict the conformational changes occurring during PPIs. Understanding these changes is crucial for elucidating the physiological effects of binder proteins during PPIs, which is a critical step in developing physiologically active protein binders (Rödström *et al.* 2024, Rustamov *et al.* 2024). These limitations underscore the need for improved methodologies to accurately capture the dynamic conformational landscapes involved in protein interactions.

Our hypothesis is that by sampling MSA, AF-Multimer could predict the conformational selectivity and mechanistic effects of different ligands on metamorphic proteins. We aimed to demonstrate that reconstructing the conformational landscapes of proteins enables AF-Multimer to predict the favorable binding conformational states for chemokine receptor 4 (CXCR4) protein agonist (CXCL12) and antagonist (vMIP-II), as well as the glucagon receptor (GCGR) negative allosteric modulator (RAMP2). Our findings suggest that in the presence of agonists, receptor structures with high confidence, ranked by AF2’s predicted local distance difference test (plDDT), were predominantly sampled in the active state. On the contrary, in the presence of inhibitors, receptor structures were mostly predicted in the inactive state. We further explored the ability of this approach to describe the mechanistic changes occurring during ligand binding to receptors.

We also assessed whether our approach could predict the favorable conformations for state-switching proteins during oligomerization. By sampling the metamorphic proteins’ conformational landscapes, we demonstrated that AF-Multimer, enhanced by MSA clustering, could predict dimeric conformational states of SH3 domain, thermonuclease and lympholactin, when dimeric unit of the protein is given, while sampling the dimeric state of these proteins using only monomeric MSA was impossible both with AF2 and AF-Multimer.

In summary, our study illustrates that clustering MSA sequences significantly enhances AF-Multimer’s ability to predict the conformational landscapes and mechanistic effects of ligand binding. We believe that this computational tool will provide deeper insights into PPIs, paving the way for more efficient drug discovery and development processes.

## 2 Methods

### 2.1 MSA construction and prediction

MSAs were generated using the MMseqs-based (Steinegger and Soding 2017) protocol implemented in ColabFold (Mirdita *et al.* 2022). Structure predictions based on the obtained MSAs were performed on a local ColabFold setup using a single NVIDIA RTX 3060 GPU. Default ColabFold predictions were carried out with five models and three recycles using both AF2 and AF2-Multimer-v3 models. To predict subsampled populations of the studied proteins, ColabFold’s implementation of MSA subsampling was employed with various max MSA values as described by (Monteiro da Silva *et al.* 2024).

MSA clustering and random sampling were conducted using AFcluster (Wayment-Steele *et al.* 2024). Sequences containing more than 25% gaps were excluded prior to clustering. MSAs were clustered using the Density-Based Spatial Clustering of Applications with Noise (DBSCAN) algorithm implemented in AFcluster, which identifies core density regions within the MSA and clusters sequences that have at least *k* points within a specified epsilon distance. Random sampling was performed with MSA sizes of 10 and 100.

For homooligomeric MSAs of dimeric proteins, the state was adjusted between monomeric and oligomeric forms by duplicating sequences within clustered MSAs. To predict the conformational landscapes of receptor–ligand complexes, unpaired MSAs of both receptor and ligand sequences were concatenated with diagonal padding. AF2-Multimer-v3 or AF2 models were utilized to predict all clusters, employing a single model with three recycling steps.

### 2.2 Prediction analysis

Root-mean-square deviation (RMSD) between predicted and experimentally determined structures was computed using MDAnalysis (Michaud-Agrawal *et al.* 2011). For G-protein-coupled receptors (GPCRs), the conformational binding landscape was evaluated by calculating the RMSD of the intracellular portions of TM5-TM6 relative to reference structures (Supplementary Fig. S1). Then we examined the lowest value between RMSDs to active and inactive structure to analyze the conformational state, which predicted protein obtains (Saldaño *et al.* 2022).

To test different epsilon values in the GCGR example (Supplementary Fig. S7A), we classified predictions as active or inactive if their RMSD to any state did not exceed 5.4 Å (reflecting the difference between studied reference structure segments) and if the plDDT was >70.

Conformational landscapes of lympholactin, SH3, and thermonuclease were assessed by RMSD analysis of specific residues or full structures, as detailed in Supplementary Fig. S1. As reference structures for lympholactin, SH3, and thermonuclease obtain greater RMSD deviation, we classified predicted structures as active or inactive, if the RMSD to reference structure were lower than 3.5 Å (Monteiro da Silva *et al.* 2024).

All predicted structures and trajectories were visualized using PyMOL (version 2.5.5) (Schrodinger 2015).

### 2.3 Molecular dynamics simulations

Systems involving CXCL12 and vMIP-II bound to CXCR4 were prepared using the CHARMM-GUI (Wu *et al.* 2014) interface with the Charmm36mm force field (Huang *et al.* 2017). Receptor–ligand complexes were embedded in a POPC bilayer containing 88 molecules and solvated in TIP3 water with 150 mM NaCl (Supplementary Fig. S6A). GROMACS (Abraham *et al.* 2015) was employed for all simulations, following a six-step equilibration protocol generated by CHARMM-GUI, followed by a 300 ns NPT (constant number of particles, pressure, and temperature) run (Razzokov *et al.* 2023, Nebesnaya *et al.* 2024).

Subsequent analysis of protein structure dynamics, including RMSD and root-mean-square fluctuation (RMSF), was performed using the GROMACS package. The exploration of conformational space by CXCR4 was reconstructed and compared against reference structures using the MDAnalysis toolkit (Michaud-Agrawal *et al.* 2011).

## 3 Results

### 3.1 Predicting the conformational landscape of ligands binding to CXCR4

CXCR4, also known as CD187, is an immune checkpoint protein. Its endogenous agonist, CXCL12 (SDF1), binds to the receptor and triggers an immune response by activating CXCR4 (Saotome *et al.* 2024). In contrast, vMIP-II blocks receptor activation by binding to the orthosteric site, thereby preventing agonist binding and inhibiting the immune response (Qin *et al.* 2015). Activation of CXCR4 involves conformational changes in transmembrane helices 5 and 6 (TM5 and TM6) (Hauser *et al.* 2021), which create a binding site for the G protein in the activated state. In the absence of ligands, CXCR4 remains inactive.

**Figure 1.**
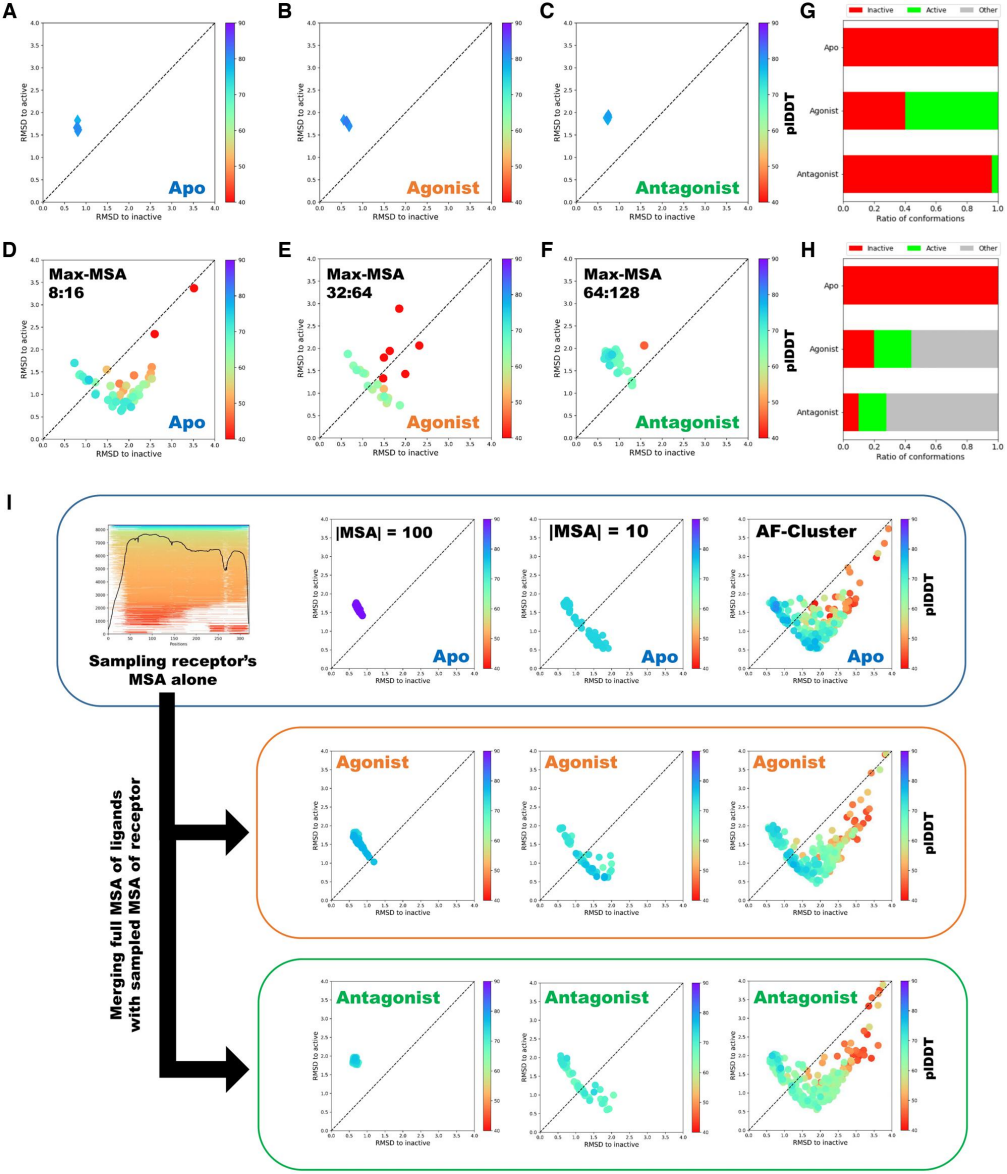
Comparison of different sampling methods in predicting the conformational landscapes of protein-protein interactions. (A–C) AF2 and AF2-Multimer predictions of CXCR4 in apo (A), agonist (B) and antagonist-bound (C) states using full-MSA yield only inactive receptor conformations. (D–F) The ColabFold implementation of random MSA subsampling can enhance the diversity of conformational states predicted by the AlphaFold2 model; however, it is highly sensitive to the max-msa parameter; (G, H) the percentage of predictions being predicted in active or inactive states using max-msa 64:128 (G) and 32:64 (H). (I) Workflow for predicting conformational landscape of protein–protein interactions using AlphaFold and unpaired sequence clustering. Initially, the MSA of the metamorphic protein is sampled or clustered using various techniques and predicted by AlphaFold2 models. Then, an unpaired MSA, comprising the full ligand MSA and the sampled/clustered MSA of the metamorphic protein, is constructed and provided as input to the AlphaFold model.

We began by investigating the effects of CXCR4 ligands on the receptor’s conformational changes. First, we predicted the structures of CXCR4 in its apo state (without ligands) and bound to its agonist (CXCL12) and antagonist (vMIP-II) using a full MSA and default AlphaFold settings. Previous studies have shown that AF2 predictions for multistate proteins are biased toward one conformational state (Saldaño *et al.* 2022). Consistent with this, our predictions using ColabFold (Mirdita *et al.* 2022), showed that (i) AF2 predicts CXCR4 only in the inactive state and (ii) AF2-Multimer does not capture the effects of CXCR4 ligand binding on conformational changes, as it predicts both agonist- and antagonist-bound receptors in the inactive conformation (Fig. 1A–C).

The first issue has been recently solved by the application of different MSA sampling methods (Monteiro da Silva *et al.* 2024, Wayment-Steele *et al.* 2024). We hypothesized that, by predicting the full PPI landscape of CXCR4–ligand interactions, we could better assess ligand selectivity and the mechanistic effects of these ligands. To test this, we predicted structures of CXCR4 in various states using ColabFold’s full MSA sampling implementation with different maximum depths for MSA subsampling, referred to as max-MSA.

We found that, with max-MSA values of 32 and 64, the AF2 model fails to capture the conformational landscape of CXCR4 in the apo state. However, setting max-MSA to eight significantly improves AF2’s ability to predict distinct conformations of CXCR4 (Fig. 1D). While AF2-Multimer predicts a broader range of CXCR4 conformational states in the presence of ligands at max-MSA values of 64 and 32, it still struggles to produce high-confidence structures of both CXCR4 conformations when bound to the antagonist (vMIP-II).

To evaluate the effects of ligands on the receptor, we measured the fractions of predicted CXCR4 conformations in both active and inactive states. Our results indicate that the predicted populations were highly sensitive to MSA depth values, especially for antagonist-bound structures, with predominantly inactive conformations at max-MSA 64 and a higher fraction of active state predictions at max-MSA 32. Furthermore, with max-MSA set to 8, AF2-Multimer was unable to predict both conformational states of ligand-bound CXCR4 (Supplementary Fig. S2).

We hypothesized that variations in structure prediction across different random sampling cutoffs are due to the more complex MSA structure for heteromers, which includes unpaired MSAs for the ligand and receptor, as well as paired MSAs for the ligand–receptor complex. Ligand binding to GPCRs primarily influences the conformational state of the receptor (as it is the metamorphic protein). The evolutionary information relevant to the receptor’s structure is represented in both the unpaired receptor MSA and the paired ligand–receptor MSA (Evans *et al.* 2022). To sample the MSA of metamorphic protein we sought two main ways: (i) clustering the paired MSA containing both receptor and ligand information, then running AF-Multimer on the clustered MSA; and (ii) clustering the unpaired MSA of the receptor, obtaining the receptor’s conformational landscape, merging it with the full unpaired MSA of the ligand, and then predicting the complexes with AF-Multimer.

First, we sampled the paired MSA of ligand-bound CXCR4 using ColabFold’s MSA subsampling implementation with different max-MSA values (Monteiro da Silva *et al.* 2024) and clustered the paired MSA using AFcluster (Wayment-Steele *et al.* 2024). However, both methods generally failed to predict both conformational states of CXCR4 in the ligand-bound state (Supplementary Fig. S3).

Next, we focused on predicting the conformational landscape of CXCR4 by sampling the receptor’s MSA alone. After this, we predicted the conformational space of ligand–receptor complexes by constructing an unpaired MSA by merging the full MSA of the ligand with the sampled MSAs of the receptor (Fig. 1I). To achieve this, we performed uniform sampling on the receptor MSA at depths of 10 and 100 and clustered the receptor MSA using AFcluster. Our results showed that AF2 failed to predict both states when uniformly sampling with an MSA size of 100, and AF-Multimer was also unable to predict both conformational states of ligand-bound CXCR4 using this approach.

While, clustering the unpaired MSA of the receptor alone with AFcluster or randomly with smaller MSA size of 10 significantly improved the ability of AF2 to sample both conformational states of apo CXCR4 with high confidence (measured by AF2’s predicted local distance difference test, plDDT). We then merged the unpaired MSAs of the ligands with the clustered or sampled receptor MSAs and predicted the conformational landscape of CXCR4 bound to its ligands. In the presence of ligands, AF-Multimer successfully sampled both conformational states of CXCR4 with high confidence (Fig. 1I). This allowed us to explore whether the resulting conformational landscapes could elucidate the binding mechanisms and actions of the selected ligands on CXCR4.

### 3.2 AFcluster-Multimer predicts the conformational binding landscape of CXCR4 ligands

**Figure 2.**
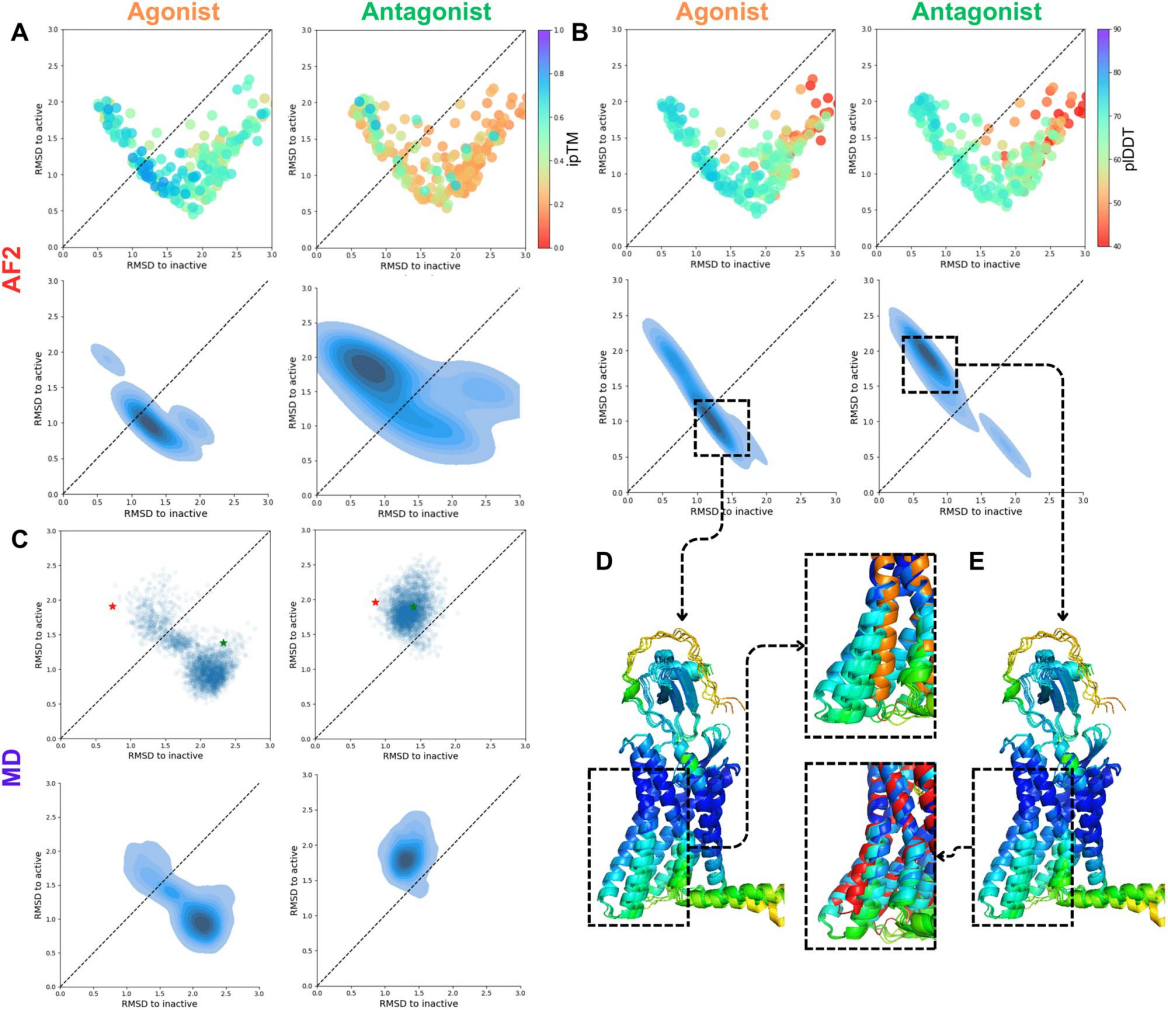
AF-Multimer combined with MSA clustering predicts conformation selectivity for CXCR4 agonist (CXCL12) and antagonist (vMIP-II). (A) TM5–TM6 RMSD of AF-Multimer predictions for all clusters to active or inactive conformations, colored by ipTM in agonists (upper left) and antagonists-bound states (upper right); distribution of top 10% predictions (ranked by ipTM) for agonist (lower left) and antagonist (lower right) bound CXCR4. (B) TM5–TM6 RMSD of AF-Multimer predictions for all clusters to active or inactive conformations, colored by plDDTs in agonists (upper left) and antagonists-bound CXCR4 (upper right); distribution of top 10% (ranked by plDDT) predictions for agonist (lower left) and antagonist (lower right) bound CXCR4. (C) Results of 300 ns MD simulations showing the conformational space exploration by agonist (left) and antagonist (right) bound CXCR4 in a POPC membrane (starting position illustrated by a red star, final position by green). (D, E) Overall structure of top 5 (ranked by plDDT) predictions for agonist-bound (D) and antagonist-bound (E) CXCR4 with a zoom on TM5–TM6 conformational changes during receptor activation [reference active structure (8U4P) shown in E, reference inactive structure (3OE6) shown in D].

To gain a comprehensive understanding of the binding effects of selected ligands on CXCR4, we tested their ability to bind to diverse conformational populations of CXCR4. We found that AF2-Multimer accurately predicts the binding orientation of CXCR4 ligands to the receptor, showing strong similarity to experimentally resolved structures (Qin *et al.* 2015, Saotome *et al.* 2024). Both CXCL12 and vMIP-II bind to the receptor in its extracellular region, with the ligand’s N terminus inserted into the receptor’s active site and the receptor’s N-terminal interacting with the ligand’s core (Fig. 2D and E), as suggested by previous computational studies (Urvas *et al.* 2024). To further investigate ligand selectivity and binding mechanisms on CXCR4, we analyzed various AF2 scoring metrics for the resulting structures.

First, we assessed the ligand binding selectivity to specific conformational states of CXCR4 by using the global binding score ipTM (interface predicted TM-score), which indicates a high probability of PPI when the score exceeds 0.5 (Martin 2024).

Our analysis of the predicted CXCR4 structures revealed a distinct shift in the conformational states of the receptor populations. Among the agonist-bound receptor structures, 18% were predicted in the inactive state, while 75% were predicted in the active state. In comparison, for the antagonist-bound structures, 29% were predicted in the inactive state, and 65% were predicted in the active state (structures with RMSD over 3 Å to any conformation were excluded from analysis). Further analysis indicated that this shift in predicted conformations became more pronounced when focusing on the predictions with the highest AF2 metrics (Supplementary Fig. S1). Specifically, 19 out of the top 23 predictions (top 10% ranked by ipTM) for the agonist-bound CXCR4 were in the active state, while 14 out of 23 predictions for the antagonist-bound receptor were in the inactive state (Supplementary Fig. S5).

We also compared the distribution of the top 10% of predictions, finding that they were densely situated in the active state for the agonist-bound receptor and in the inactive state for the antagonist-bound CXCR4. This indicates a higher binding affinity of the agonist for the active conformation of the receptor and antagonist’s higher affinity for the inactive conformation (Fig. 2A). These results illustrate the binding selectivity of ligands toward specific conformational states of the receptor.

Next, we examined the effect of ligands on the stability of receptor structures using the AF2 plDDT score, which is correlated with protein stability (Roney and Ovchinnikov 2022). In the agonist-bound state, the top 10% of predictions (ranked by plDDT) were in the active state, while antagonist-bound CXCR4 was in the inactive state (Fig. 2B).

Afterward, we aimed to measure the conformational changes in CXCR4 in the presence of its ligands using MD simulations. For this purpose, we constructed a system containing the CXCR4 receptor embedded in a POPC bilayer and bound to either the agonist or antagonist (Supplementary Fig. 6A and B). We then assessed the conformational changes occurring in the receptor during the simulation (Supplementary Fig. S6C–L). The MD simulation results showed that in the presence of the agonist, CXCR4 predominantly changed its structure to the active conformation (with total RMSD of receptor around 0.5 nm and the most significant RMSF at TM6 region) with increased TM2–TM6 distance to 2.2–2.8 nm, whereas in the presence of the antagonist remained inactive (RMSD of receptor were on the level of 0.3 nm), preserving the distance between TM2 and TM6 around 1.6 and 2.2 nm (Fig. 2C, Supplementary Fig. S6D–F). The distribution of the highest-scored ligand-binding predictions by both ipTM and plDDT closely resembled the results of a 300 ns MD simulation of ligand-bound CXCR4 (Fig. 2A–C).

These findings demonstrate that ligands exhibit conformational selectivity for receptor states, and their binding to CXCR4 leads to conformation-selective stabilization of the receptor structure. CXCL12 has a higher affinity for the active conformation of the receptor, enhancing the stability of this state and facilitating G-protein binding on the intracellular side, thereby initiating the signaling pathway. On the other hand, the antagonist vMIP-II has a higher affinity for the inactive conformation of the receptor and stabilizes this state, preventing G-protein binding and subsequent signaling.

Overall, our results show that AF-Multimer, using clustered MSAs, can effectively elucidate the mechanisms of action of CXCR4 ligands, providing valuable insights into ligand–receptor interactions and conformational dynamics.

### 3.3 AFcluster-Multimer predicts the mechanism of negative allosteric modulation of GCGR by RAMP2

In the second set of experiments, we investigated the ability of AFcluster-Multimer to elucidate the mechanism of negative allosteric modulation of the GCGR by receptor activity-modifying protein 2 (RAMP2), a mechanism not previously resolved experimentally. RAMP2 is a single-pass transmembrane protein with a C-terminal transmembrane helix bundle, that can bind to class B GPCRs and modify their activity. Specifically, it negatively modulates GCGR activity; however, the precise mechanism remains unknown (Krishna Kumar *et al.* 2023). Thus, we implemented AF-Multimer with clustered MSA to understand the mechanisms underlying RAMP2's negative modulation of GCGR.

GCGR is a class B GPCR with an extracellular N-terminal domain (Zhang *et al.* 2017). Previous studies have shown that AF2 predominantly predicts the active state conformation for class B GPCRs (He *et al.* 2023). Indeed, the default AF2 run on ColabFold returned GCGR in an active conformational state. Therefore, we used AFcluster to design different conformational states of GCGR and reconstruct the binding landscape of RAMP2 to this receptor.

First, we clustered the MSA of GCGR using AFcluster to sample both the active and inactive states of the receptor without its ligands. However, AFcluster initially failed to sample the active state of GCGR, and predicting all MSA clusters with very low plDDT and pTM scores (Supplementary Fig. S7A). In AFcluster, DBSCAN identifies cluster sizes using the epsilon parameter, which controls the maximum allowable distance for joining samples in sequence space. Higher epsilon values join more sequences into clusters, resulting in fewer clusters (Wayment-Steele *et al.* 2024). We hypothesized that the default epsilon value (which in AFcluster is the value that returns the maximum number of clusters: epsmax) for GCGR resulted in very small clusters, leading to lower AF2 prediction confidences. Therefore, we experimented with different epsilon values higher than epsmax. We found that increasing the epsilon value allowed AF2 to predict both conformational states with higher confidence as well as an increased ratio of predictions being in active state, although the total number of clusters significantly dropped. We found that clustering with an epsilon value of 13 enabled predicting the maximum number of clusters being in active or inactive conformations and plDDT score higher than 70 (Supplementary Fig. S7B). We used this epsilon parameter for our further investigation of GCGR’s conformational landscape with and without ligands.

Using an epsilon value of 13, we predicted the binding landscape of RAMP2 to GCGR. We found that RAMP2 binds to the extracellular domain of GCGR with its N-terminal domain, while its transmembrane helix bundle binds to TM5-TM6, the part of the receptor responsible for activation. The presence of RAMP2 significantly improved the overall confidence of the GCGR structure (measured by the AF2 plDDT score) compared to the apo state. Our results showed that RAMP2's presence improved AF2 confidence for all GCGR transmembrane helices (Supplementary Fig. 8).

**Figure 3.**
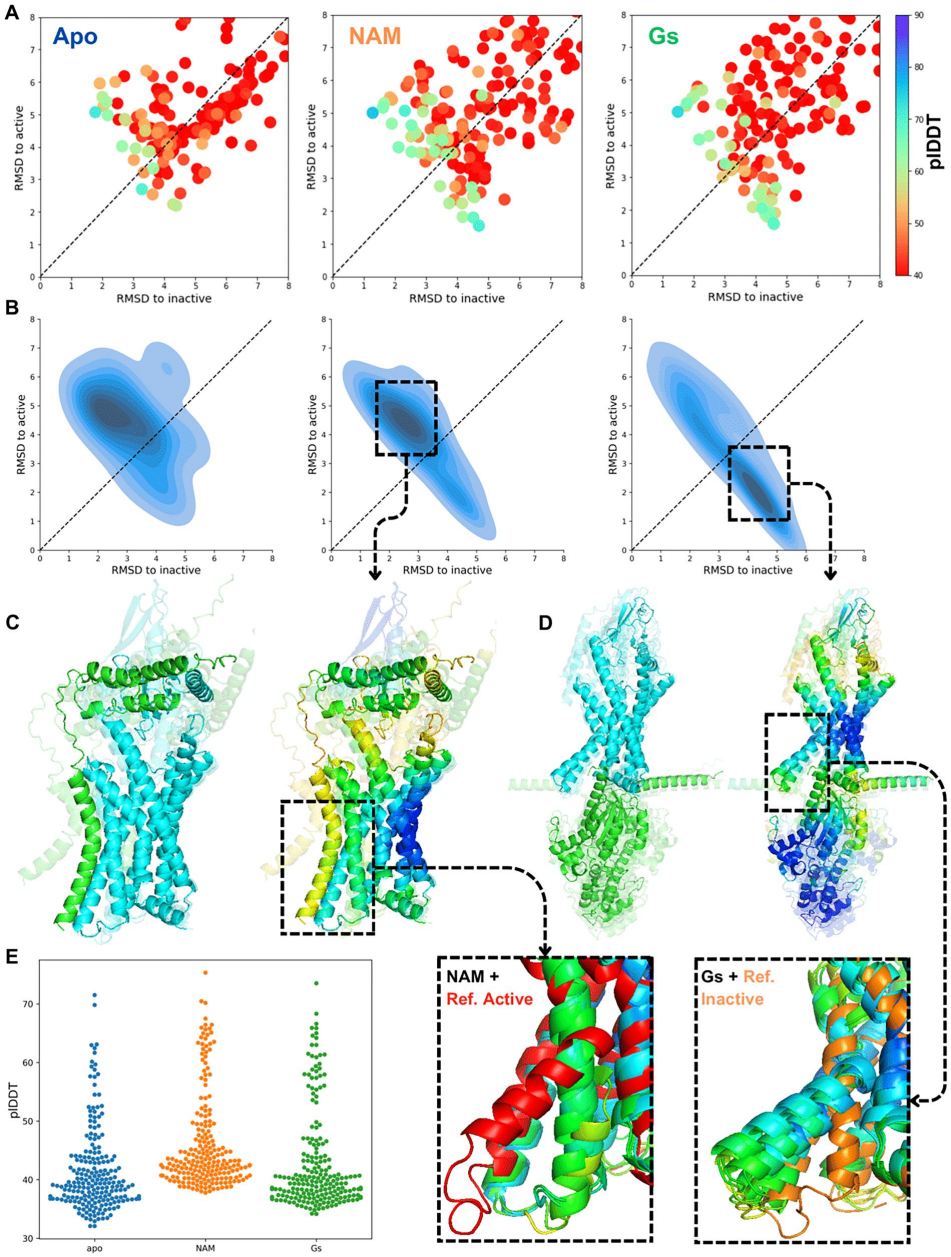
AFcluster predicts the conformational selectivity for RAMP2 and Gs binding to GCGR. (A, B) plDDTs and distribution of top 10% predictions (ranked by plDDT) for apo (left), RAMP2-bound (center), and Gs-bound (right) GCGR. (C, D) Top 5 predictions for RAMP2-bound (C) and Gs-bound (D) GCGR, colored by chain (left) and plDDT (right) with zoom-in of TM5-TM6 region compared to reference active structure and inactive structures. (E) Overall plDDTs of GCGR in apo, RAMP2-bound, and Gs-bound states.

We analyzed the binding landscape of RAMP2 to GCGR and found that the distribution of the top 10% of receptor predictions (ranked by plDDT) in the presence of RAMP2 was shifted toward the inactive state (Fig. 3B). RAMP2 binding to the ligand-binding domain of GCGR blocks the natural ligand’s access, inhibiting GCGR activity. Additionally, we found that RAMP2’s transmembrane domain binds to the TM5–TM6 interface of GCGR, stabilizing the receptor structure in the inactive state (Fig. 3C). This finding elucidates the mechanism of RAMP2’s negative allosteric modulation of GCGR.

Then we predicted the conformational landscape of GCGR natural ligand glucagon to the receptor. We merged glucagon’s full MSA with receptor MSA clusters and sampled ligand–receptor conformations using AlphaFold-Multimer. We found that in the presence of glucagon, top 10% of receptor predictions (ranked by plDDT) was mainly shifted toward the active state. However, analyzing the binding poses of glucagon binding to GCGR we found that in the inactive state, the glucagon was predicted similar to the crystal structures, binding to the extracellular part of receptor, while for the active state receptor population the binding pose was mainly wrong, with most of the predictions depicting glucagon bound to the intracellular part of receptor in the site of G-protein binding. The wrong binding poses of glucagon to GCGR by AF2 shows the main limitation of our approach in predicting the mechanistic effects of ligands to receptor proteins (Supplementary Fig. S9).

It has been shown that AF2 predominantly predicts GPCRs in an active state in the presence of G protein (He *et al.* 2023). We predicted the conformational space of GCGR in the presence of Gs protein. First, we predicted the full GCGR complex in the presence of Gs using AF2 and full unpaired MSA. Using full-length unpaired MSA, AF-Multimer predicted GCGR in an inactive state, with Gs located in the extracellular ligand-binding part of GCGR instead of its intracellular G-protein-binding site (Supplementary Fig. S10). We then applied our method to predict the binding conformational landscape of the Gs protein to GCGR (Fig. 3). The distribution of the top 10% of predictions (based on plDDT) was shifted to the active state (Fig. 3B). Sampling GCGR–Gs complex structures in the region with the highest plDDT distribution returned the correct binding pose of Gs and GCGR (Fig. 3D), correlating with the receptor’s active state.

These results demonstrate that AFcluster-Multimer can effectively predict the mechanisms of negative allosteric modulation and provide insights into the conformational dynamics and binding landscapes of GPCRs and their modulators.

### 3.4 Predicting the oligomerization conformational landscape

Previously it has been shown that AFcluster can successfully predict multiple states of various metamorphic proteins. However, Wayment-Steele *et al.* highlighted a limitation of AFcluster to predict the oligomeric states of proteins that switch between monomeric and oligomeric conformations (Wayment-Steele *et al.* 2024). To address this challenge, we tested our approach on metamorphic proteins, which switch between monomeric and dimeric states during physiological processes. We began by clustering the MSA for monomer subunits using AFcluster to predict the conformational landscape of the protein in its monomeric form. Subsequently, we duplicated the number of protein copies in each MSA cluster to predict the dimeric form, thus obtaining its oligomerization conformational landscape.

**Figure 4.**
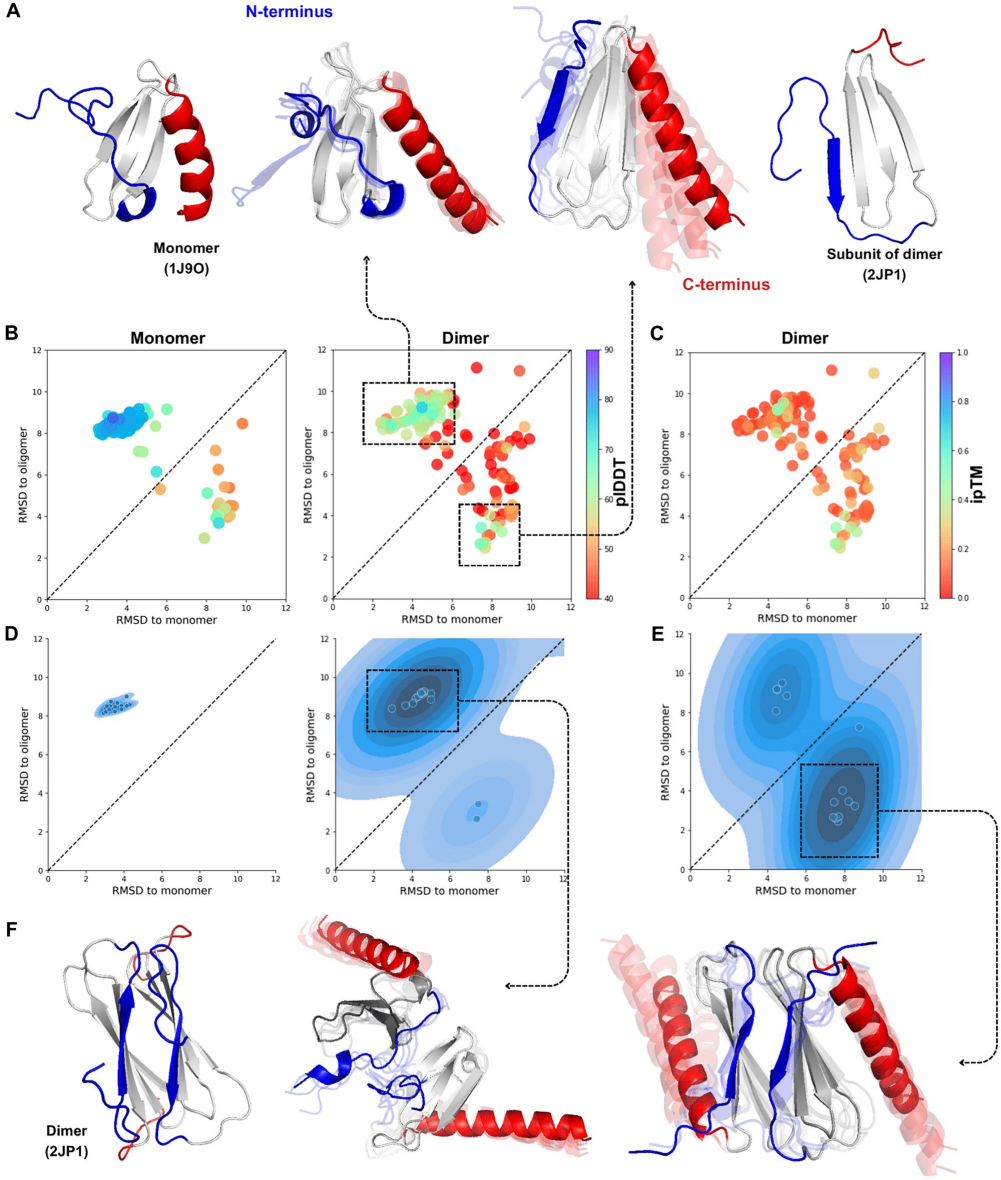
AFcluster predicts fold switching for human lympholactin N terminus in the dimeric state. (A) Reference monomer and dimeric experimental structures for human lympholactin. (B) Conformational landscape for lympholactin in monomeric (left) and dimeric (right) forms, colored by plDDT. (C) ipTMs for AF-Multimer predictions of lympholactin dimer MSA clusters. (D) Distribution of the top 10% (ranked by plDDT) of AF-Multimer predictions for monomer (left) and dimer (right) lympholactin structures. (E) Distribution of the top 10% (ranked by ipTM) of AF-Multimer predictions for the dimer. F) Subunits orientation in dimeric lympholactin in the experimental structure, compared to predicted results close to monomeric (center) and dimeric states (right).

We first examined the conformational landscape of the human lympholactin protein in both monomeric and dimeric states. Lympholactin switches from a closed state in its monomer form to an open state during dimerization (Tuinstra *et al.* 2008). When predicting lympholactin in its monomer form, AFcluster only sampled the monomeric form with high confidence (Fig. 4B and D), as previously studied. However, predicting lympholactin in its dimeric form allowed AFcluster-Multimer to sample a wider range of conformations. Despite this, the most stable forms of lympholactin’s C terminus remained in an alpha-helix conformation, indicative of the monomer form, while AFcluster successfully predicted the dimeric form of lympholactin’s N terminus (beta-sheet) with high confidence. Notably, AFcluster-Multimer achieved the highest ipTM scores for the lympholactin dimer in its N terminus dimeric conformation.

We observed that default AF-Multimer predictions for lympholactin dimers resulted in incorrect complex structures (Supplementary Fig. S12). Predictions sampled in the monomeric conformation also yielded incorrect interaction structures. However, dimer predictions sampled from regions with the highest ipTM distribution (dimeric state) produced the correct dimeric structure (Fig. 4F).

We extended this approach to other proteins, including the mouse ESH8 SH3 domain (Kishan *et al.* 2001) and staphylococcal thermonuclease (Green *et al.* 1995), both of which switch between a closed monomeric state to an open dimeric state via domain swapping. This process involves the exchange of identical structural elements between subunits, which leads to the stabilization of the open conformation of one protein molecule by the second oligomeric protomer (Bennett *et al.* 1995).

**Figure 5.**
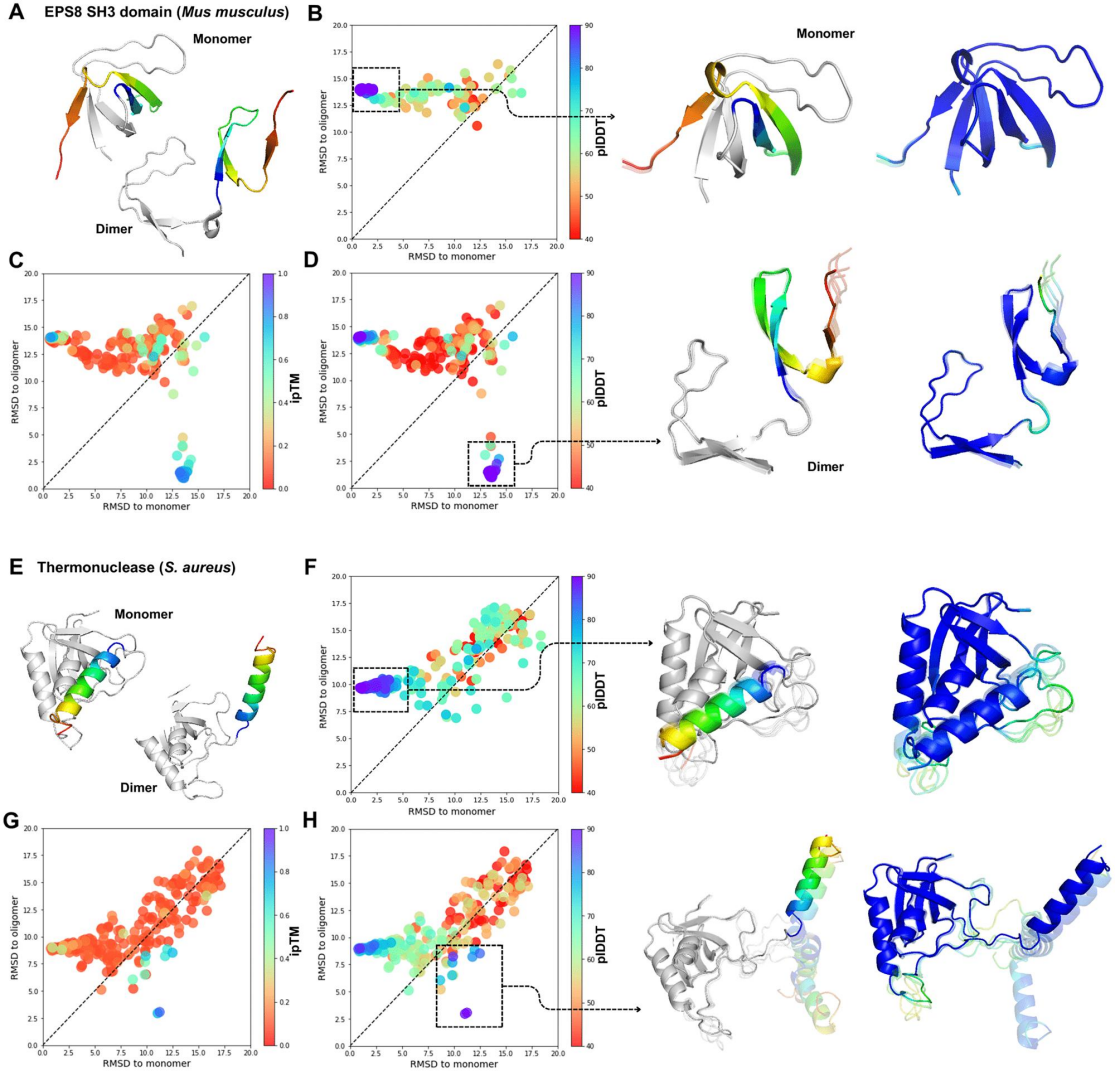
AF-Multimer predicts the oligomeric open states for SH3 domain and thermonuclease. (A) Mice SH3 domain obtains close conformation in monomer state and open state during the dimerization; (B) RMSD for AF2 predictions of all clusters to open and closed conformations (colored by plDDT); (C, D) conformational landscape of AF-Multimer predictions of all clusters in dimer state colored by ipTM (C) and plDDT (D); (E) thermonuclease obtains close conformation in monomer state and open state during the dimerization; (F) RMSD for AF2 predictions of all clusters to open and closed conformations (colored by plDDT); (G, H) conformational landscape of AF-Multimer predictions of all clusters in dimer state colored by ipTM (C) and plDDT (D).

When predicting the conformational landscape of the monomer SH3 domain, AFcluster exclusively sampled predictions in the monomeric state and failed to predict the dimeric form (Fig. 5B). In contrast, doubling the copies of MSAs for SH3 domain and predicting the dimeric structures returned both monomeric and dimeric states with very high confidence (Fig. 5D). We obtained similar results for staphylococcal thermonuclease: monomer predictions only sampled the monomeric state with high confidence (Fig. 5F), while dimer sampling predicted the dimeric conformation with high stability (Fig. 5H). Predictions with the highest plDDT scores were sampled in the dimeric conformation, and only predictions in this form achieved high ipTM scores (Fig. 5G).

In the case of SH3, where AF2 was unable to sample the dimeric conformation of the protein but AF-Multimer successfully sampled both states with high confidence, we investigated the factors contributing to this improvement. Specifically, we aimed to determine whether the enhancement was due to the presence of the dimeric unit itself or the use of the AF2-Multimer model. To explore this, we predicted the conformational landscape of SH3 in both its monomeric and dimeric forms using both AF2 and AF-Multimer. Our results showed that AF2 could not predict the dimeric conformation of SH3 when provided with a dimeric unit MSA, and similarly, AF2-Multimer was unable to sample both conformations when given a monomeric unit MSA as input (Supplementary Fig. S11). These findings demonstrate that the presence of the dimeric unit is necessary for predicting the dimeric conformation of the protein, as the second subunit likely provides energetic stabilization for the domain swapping required for dimerization.

Lympholactin, the SH3 domain, and thermonuclease all switch between monomeric and dimeric forms. AFcluster fails to predict the dimeric conformation of these proteins when predicting the monomeric form, and AF2 predicts the dimeric forms with very low confidence or not at all. This phenomenon can be attributed to the energetically unfavorable state of the dimeric conformation in the monomer subunit without stabilization by the second subunit. For the SH3 domain and thermonuclease, stabilization of the dimeric conformation likely only occurs in the presence of the second subunit, making it improbable in the monomeric form. Consequently, AF2-Multimer also fails to predict such states without the stabilizing subunit.

Our approach can be used to describe changes in the conformational space of proteins during oligomerization, offering a valuable tool for understanding the dynamics of protein assembly.

## 4 Discussion

AF2 has revolutionized the field of computational biology by providing highly accurate predictions of protein structures (Pereira *et al.* 2021). The recent advancements in MSA clustering methods have further enhanced AF2’s ability to predict different conformations of metamorphic proteins (Wayment-Steele *et al.* 2024), providing deeper insights into protein functions. In this study, we demonstrated that predicting multiple conformational states of metamorphic proteins in complex with ligands or in oligomeric states can reveal the conformational changes occurring during PPIs. Understanding the mechanistic effects of ligands on receptors has significant implications in drug design, particularly for the targeted design of binders with specific functionalities—such as modulators that activate or inhibit physiological or pathological functions of target proteins (Rödström *et al.* 2024, Rustamov *et al.* 2024).

Using CXCR4 as a model system, we found that AF2, when using a full MSA, fails to predict the effects of ligands on this receptor, as it predicts both agonist- and antagonist-bound forms in the inactive state. Although subsampling the full MSA of ligand-bound CXCR4 can increase the conformational diversity of ligand–receptor complexes, this approach still struggles to predict both conformational states with high confidence, particularly for the antagonist-bound receptor. We hypothesized that this limitation may stem from the more complex structure of the ligand–receptor MSA. To address this, we first sampled the MSA of the metamorphic protein and then stacked the MSAs of ligands onto the resulting clusters, which successfully sampled diverse conformational states of the studied proteins. This approach enabled us to uncover the conformational binding landscapes and mechanistic effects of ligands on CXCR4.

Our analysis of CXCR4 in both apo and ligand-bound states revealed the mechanism of ligand action and its conformational selectivity. We observed that AFcluster-Multimer could successfully sample structures with the highest confidence in the active conformation for agonist-bound states and in the inactive conformation for antagonist-bound states. The distribution of CXCR4 complex predictions with the highest AF2 confidence closely resembled the results of MD simulations, validating our approach as a reliable computational tool for studying ligand-induced conformational changes.

To further test the robustness of our method, we applied it to the unresolved complex of the GCGR with receptor activity-modifying protein 2 (RAMP2). By clustering the MSA of GCGR and then predicting the binding landscape of RAMP2, we found that RAMP2 binds to the extracellular domain of GCGR and its transmembrane helix bundle binds to the TM5-TM6 interface, stabilizing GCGR in its inactive state. This finding elucidates the mechanism of negative allosteric modulation of GCGR by RAMP2, which had not been experimentally resolved previously.

Additionally, we assessed the ability of our approach to predict favorable conformations for state-switching proteins during oligomerization. Our results showed that AF-Multimer combined with doubled MSA copies can predict the dimeric conformations of lympholactin, SH3 domain thermonuclease, and other metamorphic proteins with high confidence. In contrast, AF2 alone failed to sample dimeric conformations of these proteins in their monomeric form, highlighting the importance of our MSA clustering approach in capturing the oligomerization process.

Our approach is entirely based on the ability of AF2 and AF2-Multimer (AF2-M) to accurately predict ligand–receptor complexes. However, its main limitation arises when AF2 models fail to predict protein interactions, such as the binding between glucagon and the GCGR. Additionally, since AF2 models can only predict the structures of proteins, our approach cannot address the mechanistic effects of most natural and synthetic ligands, which are typically small molecules, on receptor proteins. Nevertheless, this limitation may be effectively addressed with the release in open-source of the new AlphaFold model (AlphaFold3), which is not restricted to proteins alone (Abramson *et al.* 2024).

In conclusion, our study demonstrates that clustering MSAs significantly enhances AF-Multimer’s ability to predict the conformational landscapes and mechanistic effects of ligand binding. This computational tool provides valuable insights into protein–ligand interactions and has the potential to accelerate drug discovery and development by offering a deeper understanding of protein conformational dynamics.

## Supplementary Material

vbae197_Supplementary_Data

## Data Availability

Scripts for running MSA construction, AF2 or AF-Multimer, and analysis presented here are available at GitHub (https://github.com/KhondamirRustamov/AF-Multimer-cluster).
