# Supplementary material for: MSA clustering enhances AF-Multimer’s ability to predict conformational landscapes of protein–protein interactions
Source: Bioinform Adv. 2024 Dec 6;5(1):vbae197. doi: 10.1093/bioadv/vbae197 (PMC11671036; doi:10.1093/bioadv/vbae197)
Supplement: vbae197_Supplementary_Data [file vbae197_supplementary_data.docx]

**Supplementary data for:**

**MSA clustering enhances AF-Multimer's ability to predict conformational landscapes of protein-protein interactions**

**Kh.R. Rustamov^1^*, A.Y. Baev^1,2^*.**

^1^Laboratory of Experimental Biophysics, Center for Advanced Technologies, Tashkent, Uzbekistan.

^2^National University of Uzbekistan, Tashkent, Uzbekistan.

Author for correspondence: [x.rustamov@cat-science.uz](mailto:x.rustamov@cat-science.uz), [baev.a.yu@gmail.com](mailto:baev.a.yu@gmail.com)


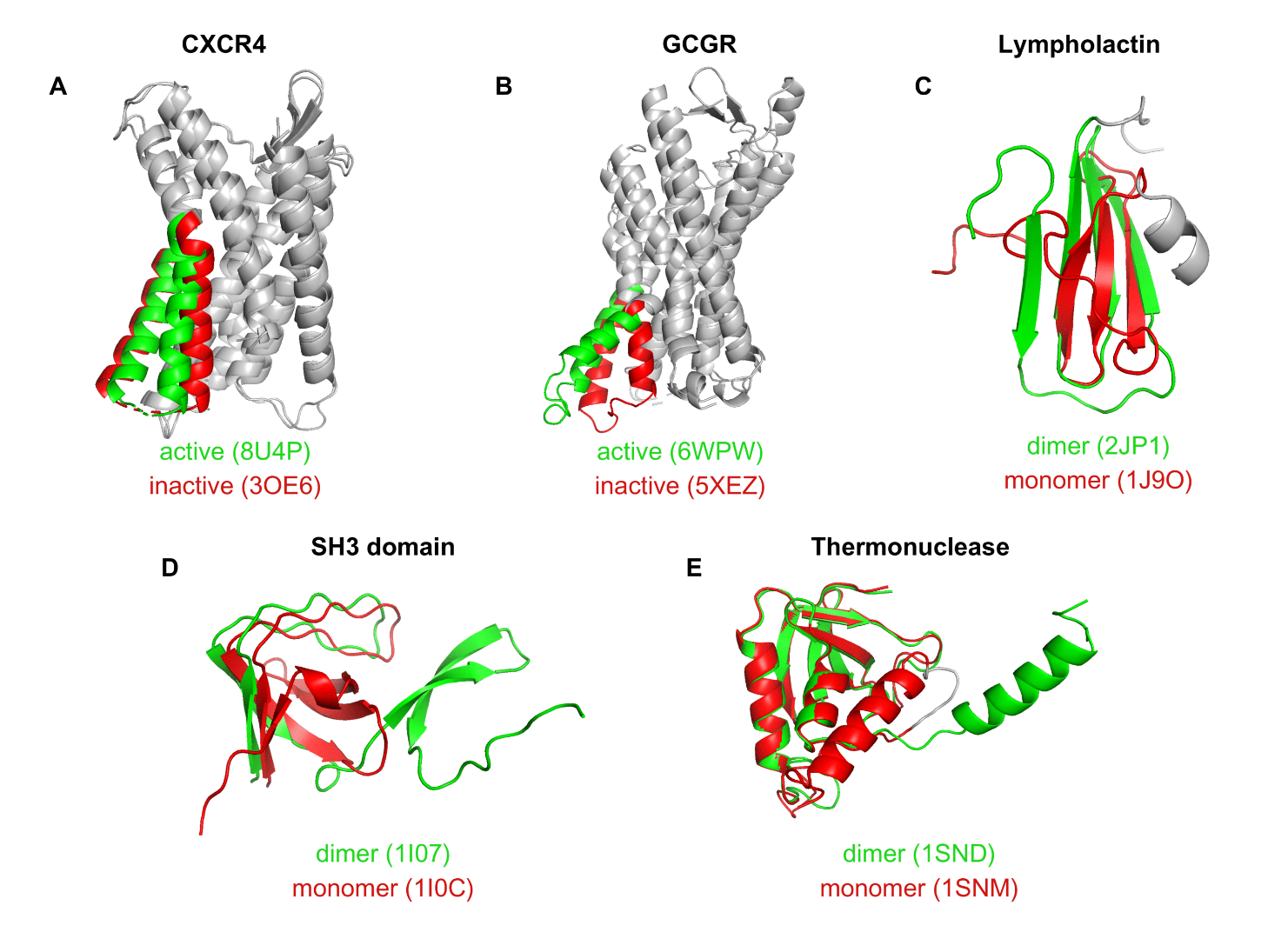


**Supplementary Figure 1. Experimental reference structures for metamorphic proteins used in this study.** **A, B)** reference structures for CXCR4 and GCGR with highlighted TM5-TM6 regions, corresponding to active-inactive conformation switches; **C)** reference structure for monomeric and dimeric human lympholactin with highlighted region used to comparative analysis (excluding the C-terminus); **D, E)** reference structures for SH3 domain and thermonuclease.


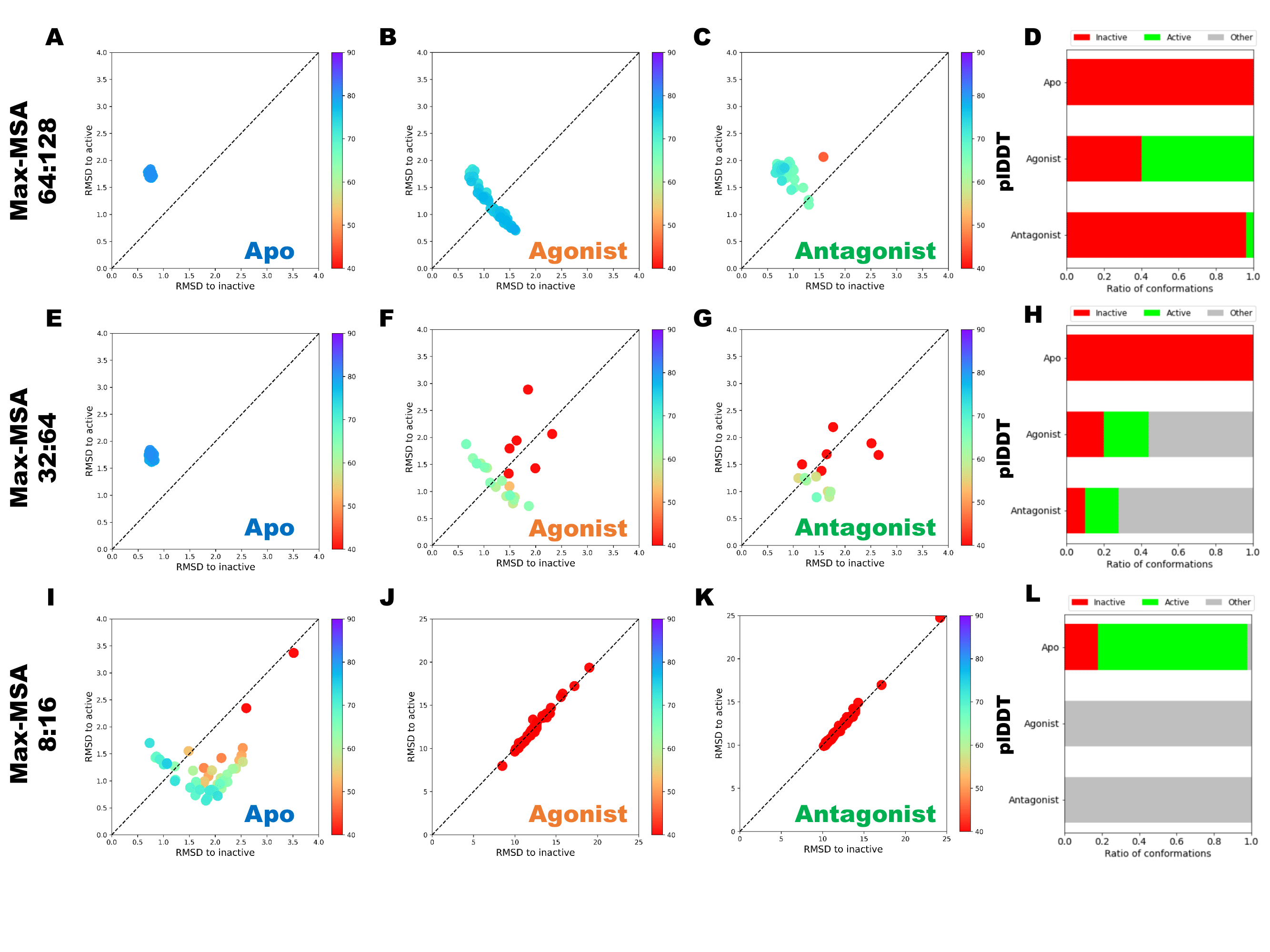


**Supplementary Figure 2. Predicting the conformational landscape of CXCR4 in apo, agonist and antagonist-bound states using MSA subsampling implementation in ColabFold. A, B, C)** conformational landscapes of apo and ligand bound CXCR4 using max-MSA 64:128; **D)** the percentage of receptor structures predicted in active, inactive conformations using MSA subsampling with max-MSA 64:128; **E, G, F)** conformational landscapes of apo and ligand bound CXCR4 using max-MSA 32:64; **H)** the percentage of receptor structures predicted in active, inactive conformations using MSA subsampling with max-MSA 32:64; **I, J, K)** the conformational landscapes of apo and ligand bound CXCR4 using max-MSA 8:16; **L)** the percentage of receptor structures predicted in active, inactive conformations using MSA subsampling with max-MSA 8:16;


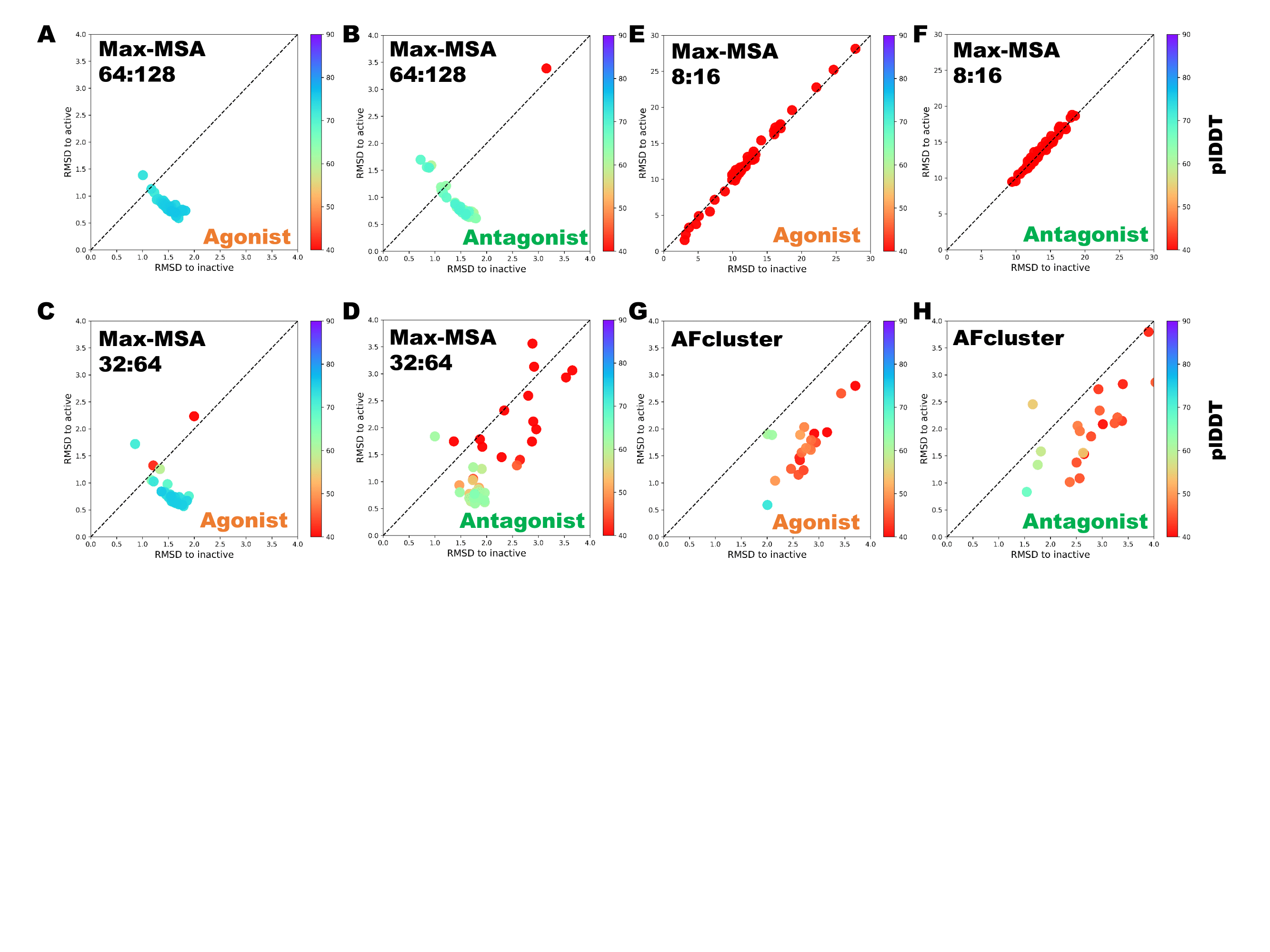


**Supplementary Figure 3. Predicting the conformational landscape of CXCR4 in apo, agonist and antagonist-bound states using subsampling of paired MSA using different methods. A, B)** conformational landscapes of apo and ligand bound CXCR4 using paired MSA and max-MSA 64:128; **C,** **D)** conformational landscapes of apo and ligand bound CXCR4 using paired MSA and max-MSA 32:64; **E, F)** the conformational landscapes of apo and ligand bound CXCR4 using paired MSA and max-MSA 8:16; **G, H)** the conformational landscapes of apo and ligand bound CXCR4 using paired MSA and AFcluster.


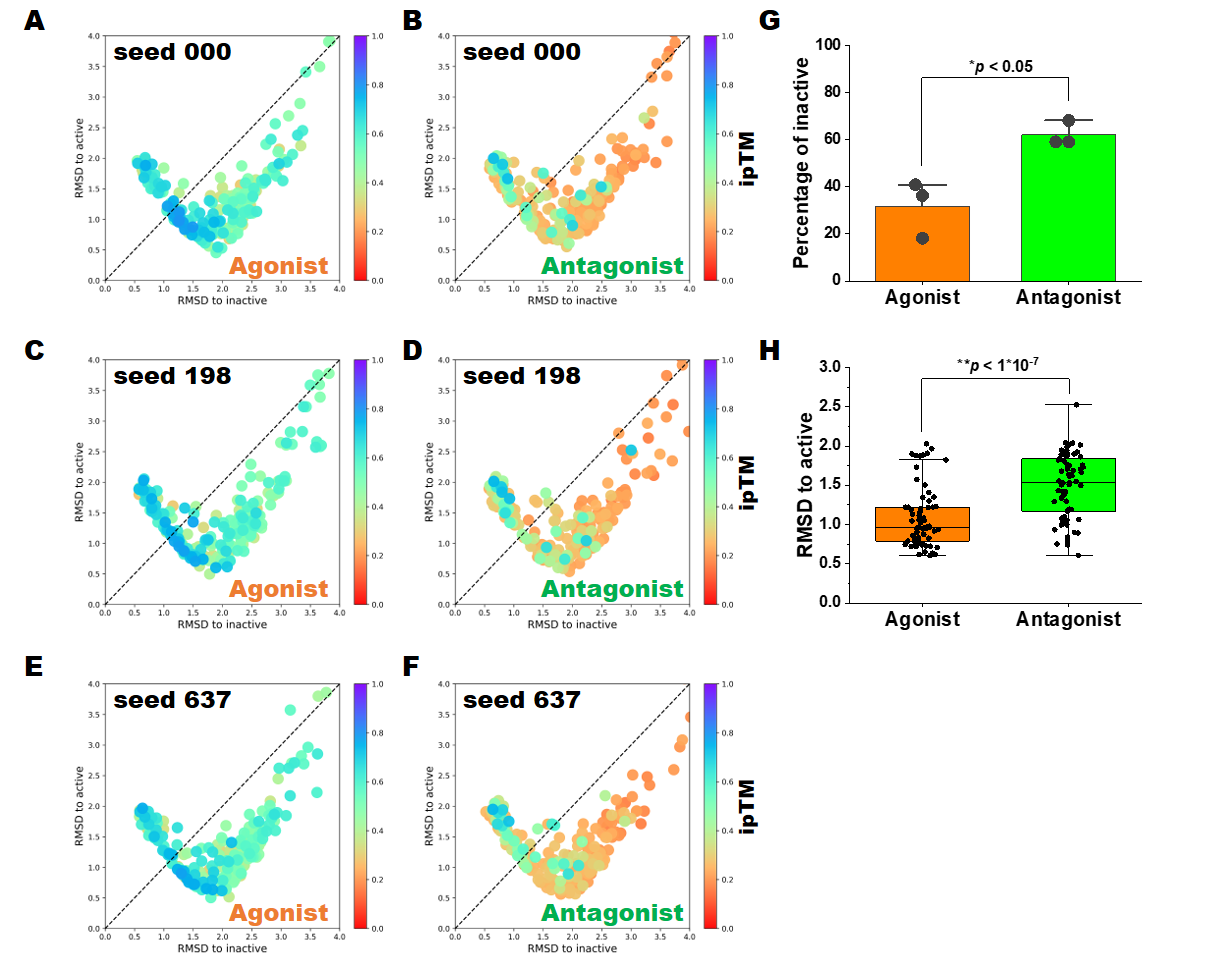


**Supplementary Figure 4. Predicting the conformational landscape of CXCR4 in agonist and antagonist-bound states using AFcluster and AF2-Multimer with different seeds.** Conformational landscapes of agonist and antagonist bound CXCR4 using AF2-Multimer seed 000 (A, B), 198 (C, D) and 637 (E, F) can sample both conformations of receptor, with shifts in top-10% predictions (ranked by plDDT) being shifted towards active conformation in agonist bound state and towards inactive state in antagonist bound state (G), **H)** RMSD to active conformation of ligand bound CXCR4 shows statistical significance of top predictions shift towards one conformation.


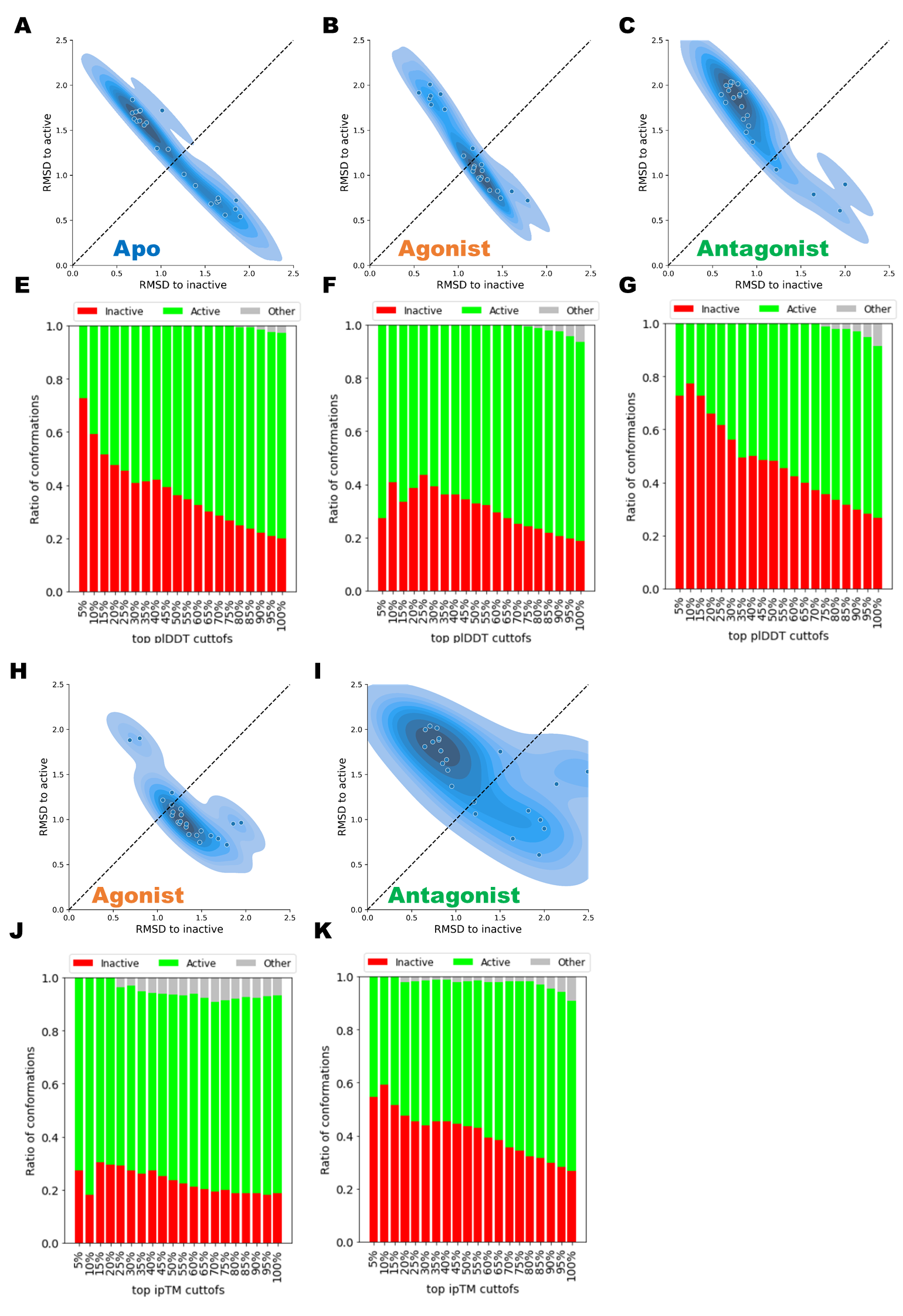


**Supplementary Figure 5. Distribution of predicted conformations being in active/inactive state with highest AF2 metrics is mainly shifted towards one state. A, B, C)** Distributions of top-10% (ranked by plDDT) predictions for CXCR4 in apo (A), agonist (B), antagonist-bound (C) state; **D, E, F)** Percentage of predictions being in active/inactive state at different plDDT cutoffs; **H, I)** Distributions of top-10% (ranked by ipTM) predictions for CXCR4 in agonist (H), antagonist-bound (I) state; J**, K)** Percentage of predictions being in active/inactive state at different ipTM cutoffs.


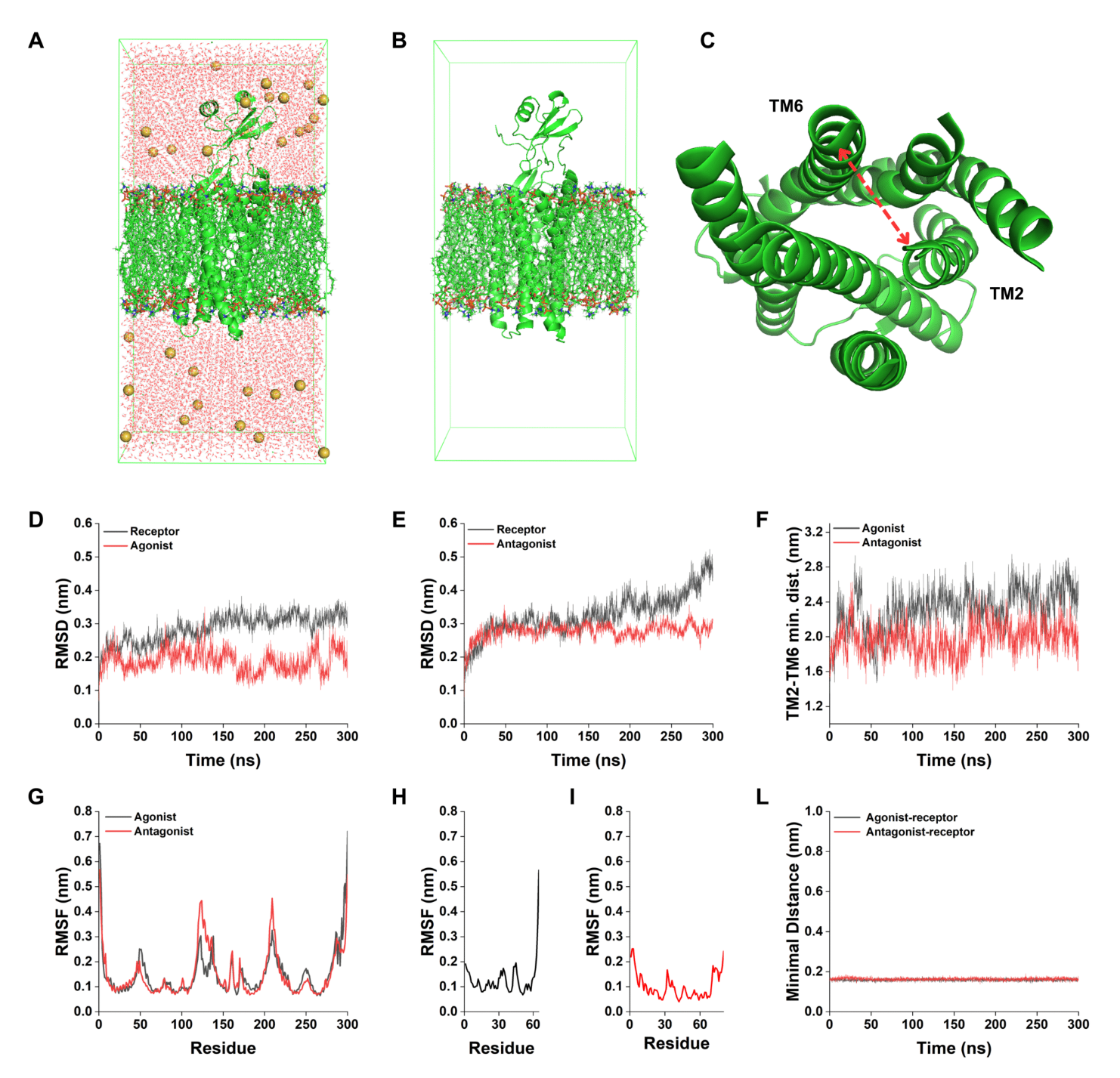


**Supplementary Figure 6. Results of MD simulations of CXCR4 bound to its ligands.** **A)** The structure of CXCR4-CXCL12 system, consisting of 88 POPC, 11261 TIP3 water molecules and 30 Na^+^ and 44 Cl^-^ ions; **B)** the structure of CXCR4-vMIP II system in POPC bilayer without water molecules and ions; **C)** view of intracellular part of CXCR4 with highlighted TM2 and TM6 helices; **D, E)** dynamics of RMSD for CXCR4 and ligands backbone atoms during the course of simulation; **F)** change in minimal distance between TM2 and TM6 during the course of simulation in agonist- and antagonist-bound CXCR4 systems; **G)** RMSF of receptor Ca atoms in agonist- and antagonist-bound states; **H, I)** RMSF of agonist (H) and antagonist (I) during the course of simulations; **L)** minimal distance between ligands and receptor during the course of MD simulations.


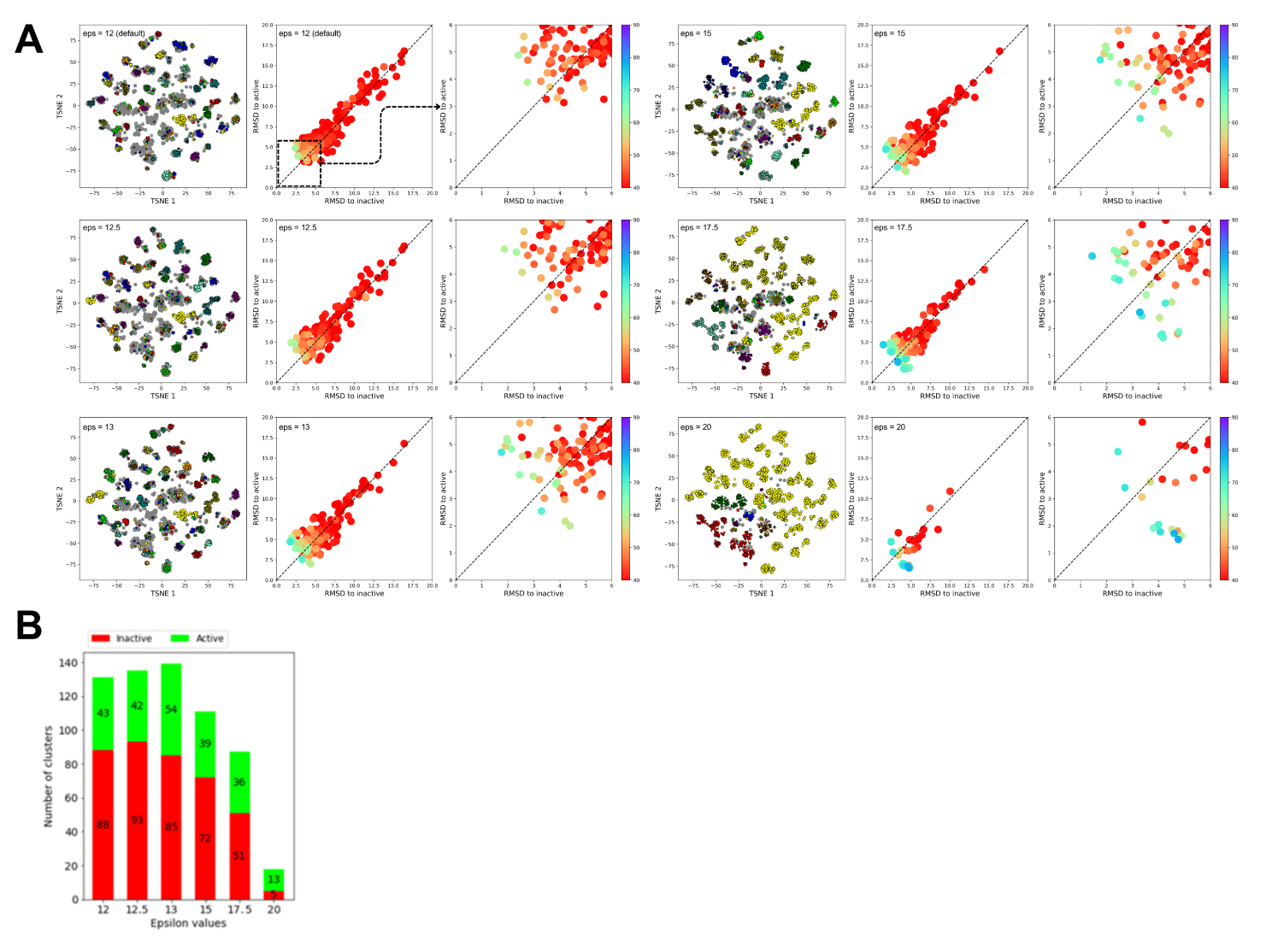


**Supplementary Figure 7. Testing different epsilon values on GCGR conformational landscape prediction enables improving of AF-Multimer structure predictions. A)** Clustered sequences (colored by clusters) and predicted structures (colored by plDDT) using AFcluster and different epsilon values; **B)** Number of clusters predicted in active or inactive conformation with plDDT exceeding 70 using different epsilon values.

**
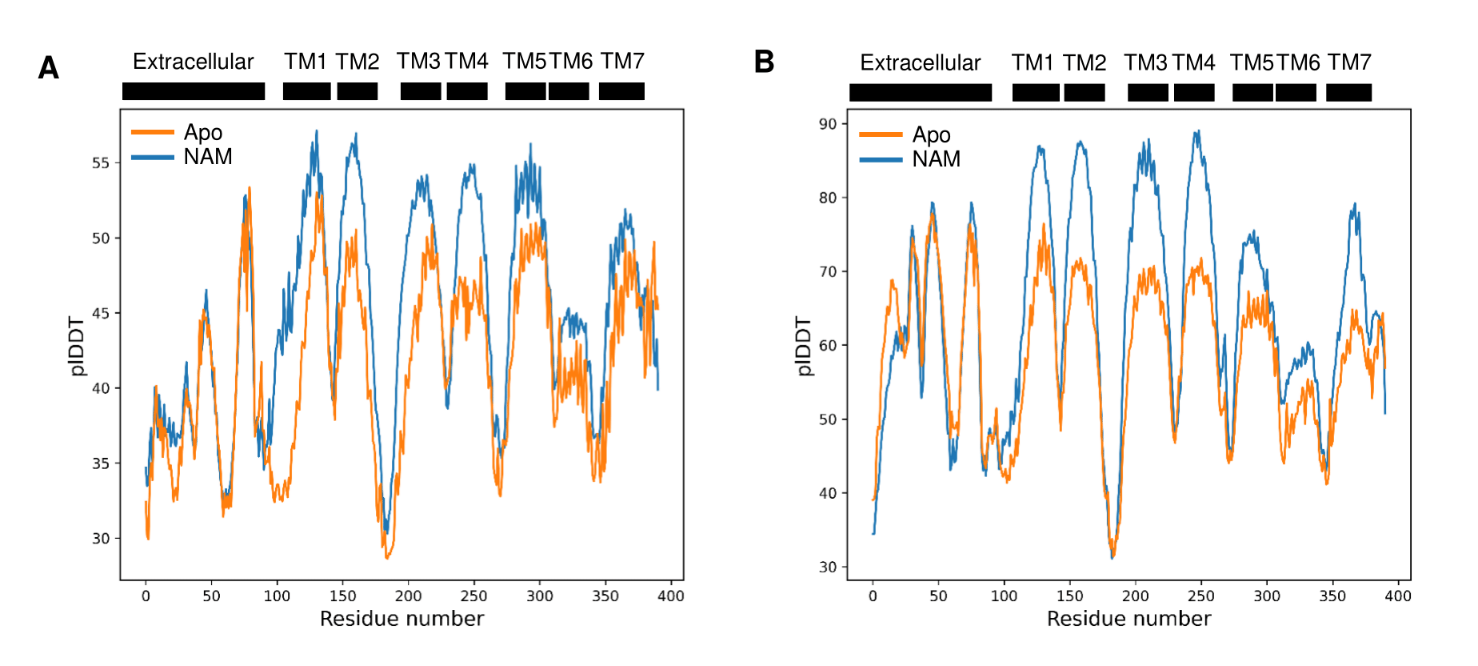
 Supplementary Figure 8. Comparison of plDDT scores for GCGR in apo and RAMP2 bound states. A)** comparison of plDDT score for mean value of all 214 GCGR predictions in apo and RAMP2 bound states; **B)** plDDT scores for mean value of top-10% GCGR predictions.


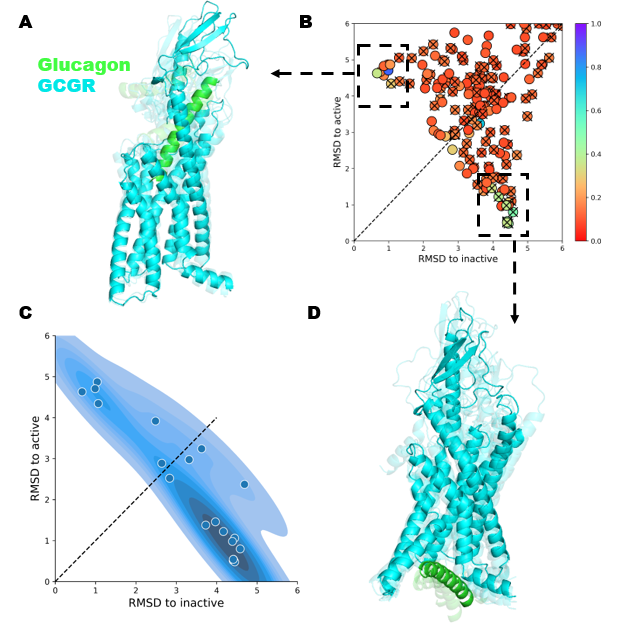


**Supplementary Figure 9. Predicting conformational landscapes of Glucagon binding to GCGR A, D)** top-5 (ranked by ipTM) predictions of Glucagon-GCGR complexes in inactive state return the correct Glucagon-GCGR binding orientation similar to experimental crystal structures (A), while top-5 predictions of Glucagon bound GCGR resulted in wrong binding state of Glucagon, with ligand bound to intracellular part of receptor (D); **B)** conformational landscape of Glucagon binding to GCGR colored by ipTM (predictions with minimal distance between Glucagon and GCGR active site residues marked with ‘x’); **C)** distribution of top-10% of predictions (ranked by ipTM) is shifted towards active state, however this is false-positive result, as AF2-Multimer cannot correctly predict the Glucagon binding orientation in receptor active state conformation.

**
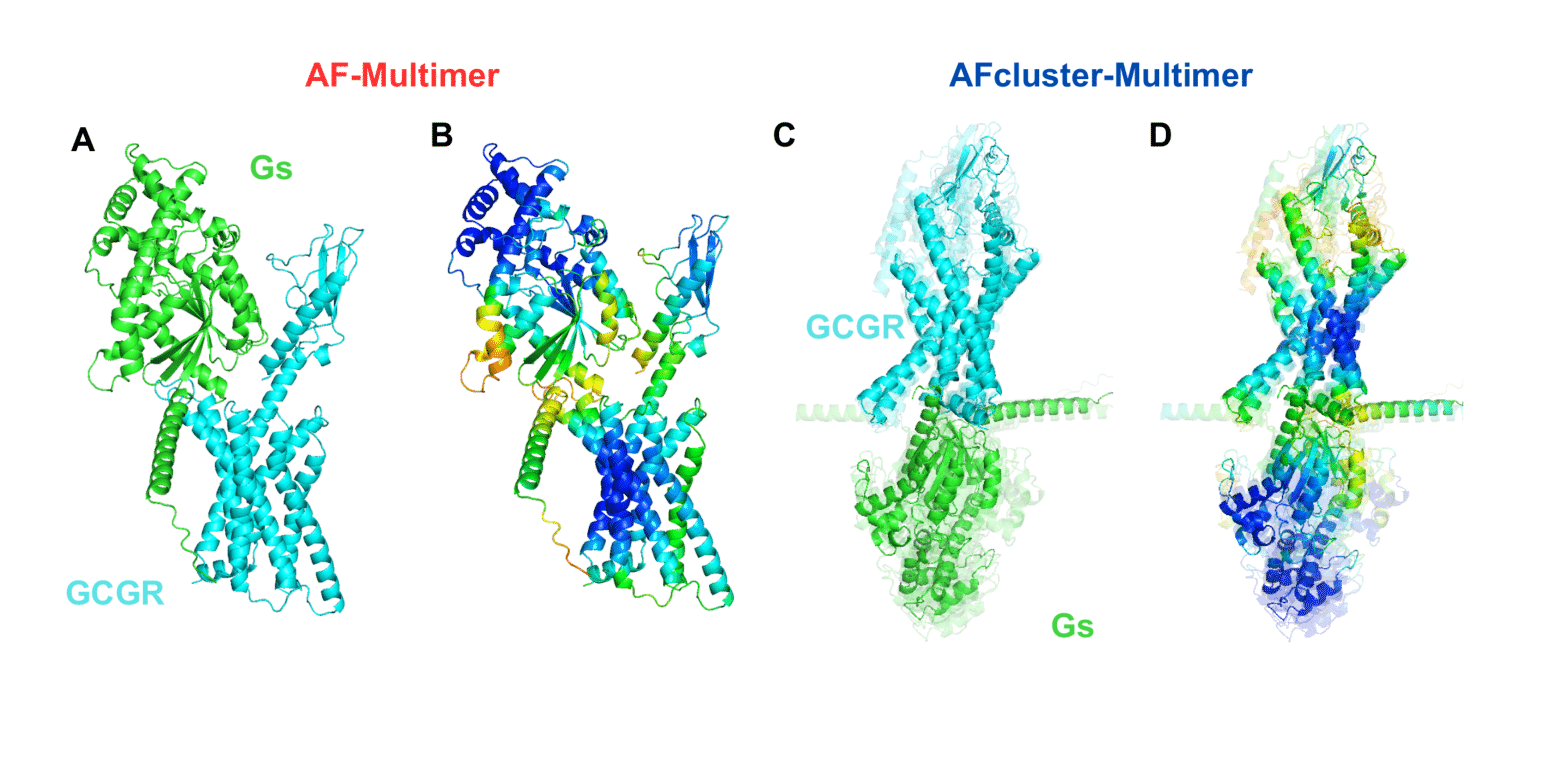
**

**Supplementary Figure 10. Sampling highest scored AFcluster-Multimer predictions enables predicting the right protein-protein interaction in GCGR-Gs complex. A, B)** the prediction of Gs-GCGR complex structures by default AF-Multimer; **C, D)** top-5 predictions (ranked by ipTM) of Gs-GCGR complex structures by AF-Multimer using subsampled MSA.


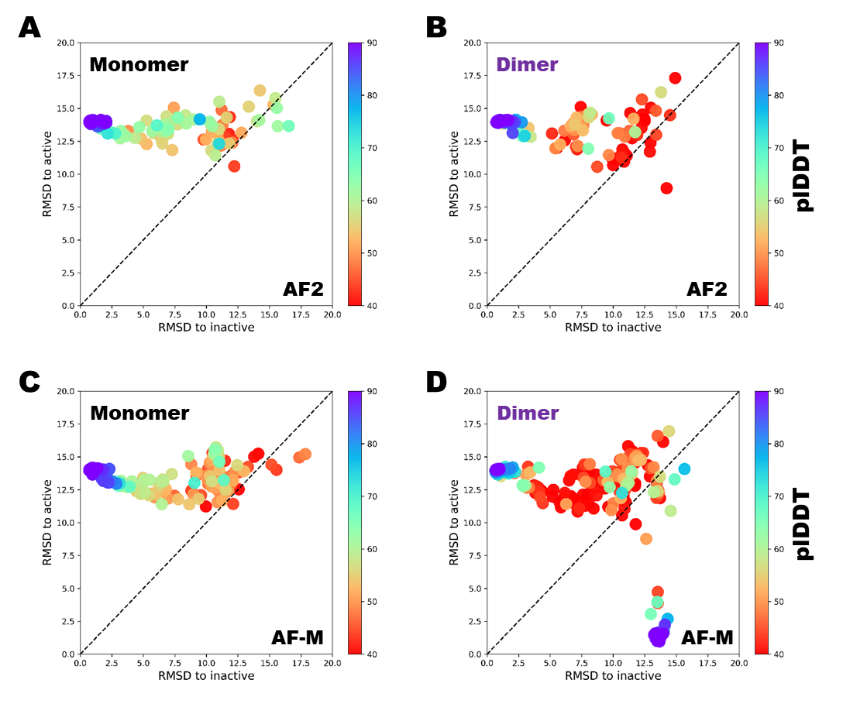


**Supplementary Figure 11. Predicting SH3 domain using AF2 and AF2-Multimer with monomer and dimer MSA units. A, B)** AF2 fails in predicting the dimeric state of SH3 domain both with one and two copies of MSA given; **C, D)** AF2-Multimer doesn’t success in sampling the dimeric conformation of SH3 with one copy of protein given as input, while successfully predicts the dimeric conformation with two copies of MSA (D).


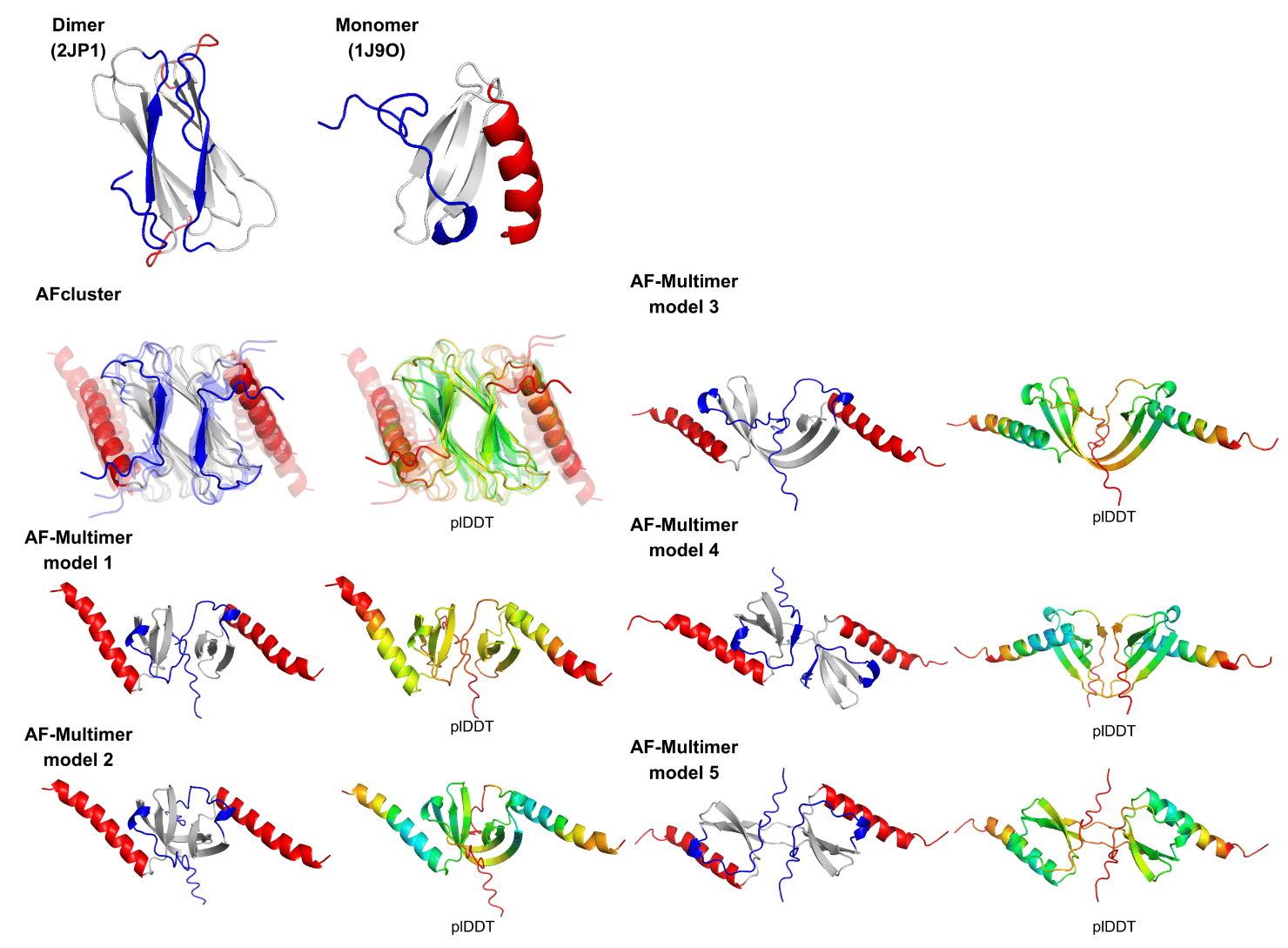


**Supplementary Figure 12. AFcluster-Multimer can successfully predict the right orientation of lympholactin dimer structure using clustered MSAs.**
